# Engineering Prostate Cancer from Induced Pluripotent Stem Cells—New Opportunities to Develop Preclinical Tools in Prostate and Prostate Cancer Studies

**DOI:** 10.3390/ijms21030905

**Published:** 2020-01-30

**Authors:** Anastasia C. Hepburn, C. H. Cole Sims, Adriana Buskin, Rakesh Heer

**Affiliations:** 1 Newcastle University Centre for Cancer, Translational and Clinical Research Institute, Paul O’Gorman building, Newcastle University, Newcastle upon Tyne NE2 4HH, UK; Cole.Sims@newcastle.ac.uk (C.H.C.S.); adriana.buskin@ncl.ac.uk (A.B.); 2 Department of Urology, Freeman Hospital, The Newcastle upon Tyne Hospitals NHS Foundation Trust, Newcastle upon Tyne NE7 7DN, UK

**Keywords:** prostate cancer, induced pluripotent stem cells, organoids, patient-derived xenografts, primary culture, cell lines, preclinical model

## Abstract

One of the key issues hampering the development of effective treatments for prostate cancer is the lack of suitable, tractable, and patient-specific in vitro models that accurately recapitulate this disease. In this review, we address the challenges of using primary cultures and patient-derived xenografts to study prostate cancer. We describe emerging approaches using primary prostate epithelial cells and prostate organoids and their genetic manipulation for disease modelling. Furthermore, the use of human prostate-derived induced pluripotent stem cells (iPSCs) is highlighted as a promising complimentary approach. Finally, we discuss the manipulation of iPSCs to generate ‘avatars’ for drug disease testing. Specifically, we describe how a conceptual advance through the creation of living biobanks of “genetically engineered cancers” that contain patient-specific driver mutations hold promise for personalised medicine.

## 1. Introduction

The development of experimental models that accurately recapitulate cancer is crucial for the study of cancer biology and development of therapeutic treatments. This is a daunting challenge given the complexity and heterogeneity seen in many cancers, including prostate cancer, which results in variation in the curative effects from person to person [1,2]. Prostate cancer is the second commonest male cancer worldwide, accounting for 1.3 million new cases and 630,000 deaths in 2018 [3]. It is an androgen-dependent disease whose growth and progression depends on the transcriptional activity of the androgen receptor (AR), also a master regulator of normal prostate epithelial cell differentiation [4]. Therapeutic options for men with localised prostate cancer include active surveillance, surgery or radiotherapy with curative intent [5,6,7,8]. For men with advanced prostate cancer, the initial mainstay for many is androgen deprivation therapy [9,10]. Despite initial favourable response, most patients progress and succumb to lethal castration-resistant prostate cancer (CRPC), the second leading cause of male cancer deaths [3,11,12]. Though next-generation hormonal treatments, such as enzalutamide and abiraterone, and chemotherapeutics, such as docetaxel, have been demonstrated to extend survival, CRPC remains a major clinical problem [13,14,15,16].

Prostate cancer is a biologically heterogeneous disease and its complex nature provides a significant challenge for its clinical management. The nature of prostate cancer heterogeneity is characterised by interpatient, intertumoural (multifocal disease), intratumoural, genomic and epigenetic heterogeneity, which raises considerable challenges when developing therapies [17]. Nevertheless, several genomic landscape studies of primary and metastatic prostate cancer have identified distinct molecular subtypes and potentially actionable genomic driver events [18,19,20,21,22]. A recurring major event is the acquired treatment resistance to hormonal approaches due to reactivation of the AR signalling pathway through AR amplification, mutations, splice variants or bypass mechanisms [21,23,24,25]. Furthermore, 10–20% of these CRPC tumours can go on to lose AR dependence altogether and exhibit small-cell neuroendocrine carcinoma characteristics (CRPC-NE) [26,27]. Therefore, development of preclinical models that can recreate this patient heterogeneity and resistance phenotypes is of upmost urgency to develop successful prostate cancer treatments.

Induced pluripotent stem cell (iPSC)-based disease modelling has proven to be a powerful tool in biomedical research and personalised regenerative medicine by improving the understanding of the disease pathophysiology of various human inherited disorders at the cellular level. The emergence of three-dimensional multi-layered organoids has attracted widespread interest and has presented a unique opportunity for high throughput drug discovery which, combined with genome editing, has become an attractive model for cancer research. This review focuses on current prostate cancer preclinical models and how recent developments and the potential manipulation of human induced pluripotent stem cells (iPSCs) could hold promise for prostate disease modelling and personalised medicine.

## 2. Challenges in Current Preclinical Prostate Cancer Models

### 2.1. Cell Lines 

A large proportion of basic cancer research today is still undertaken in cancer cell lines (Table 1). Whilst this method remains suitable for many basic research endeavours and particularly those studies focusing on molecular interactions, cell line models are not ideal for translational studies. Although many human cell lines have been propagated from clinical cancer, over time these lines accumulate multiple additional mutations that then move the genotype and phenotype away from those originally seen in the tissue from which the cells were derived [28,29]. In addition, for prostate cancer there are only a limited number of cell lines available (LNCaP, PC-3, LAPC-3, LAPC-4, VCaP. NCI-H660, MDA PCa 2a, MDA PCa 2b, CWR22Rv1, DU 145) [30,31,32,33,34,35,36,37]. Adding to the problems associated with prostate cancer cell lines is that some lack important characteristics that make their use difficult to generalise back into clinical practice. For example, PC-3 cells do not express AR [31] and NCI-H660 cells are the only line to express chromogranin A, a marker of neuroendocrine differentiation [34]. A step closer to more faithful in vitro modelling of prostate cancer may be found in using primary cultures from cells taken directly from patient tumours.

### 2.2. Primary Culture Cells

Primary benign human prostate cells can be cultured in vitro with relative ease from fresh patient biopsies. Isolating primary human epithelium (which grows from either acini structures or single cell digestions) and stroma (a mesenchymal cell mixture which supports the epithelial acini) has long been a mainstay of translational research scientists [38,39]. However, primary culture of prostate cancers is more challenging. Localised cancers are very difficult to grow, and it is those from metastatic disease that are most likely to be viable in vitro. These issues are further compounded by the small amounts of tissue available from cancer biopsies. Additionally, biopsy acquisition itself is not accessible for many researchers who lack contacts with clinical departments, and although biobanks (e.g., King’s Health Partners’ Prostate Cancer Biobank (KHP PCaBB)) are helping to provide non-clinical researchers with access to clinically relevant samples, access to fresh tissue remains difficult for most researchers [40]. In those cases where cancer primary cultures are established, ex vivo primary cells undergo spontaneous mutations in culture and deviate away from the initially derived patient genotype similar to the issue described in classical cell line models above [41]. Historically, primary prostate cells were grown in 2D and it had become recognised that their gene expression profiles and proliferation rates were different compared to counterparts grown in 3D [42]. This problem has been overcome with both 3D “tissue” like growth in xenografts and in vitro as organoids.

### 2.3. Patient-Derived Xenografts (PDXs) 

Prostate cancer-derived xenografts (PDXs) involve implantations of cells or tissue from a patient into immuno-deficient mice. The efficiency is improved with co-engraftment with mesenchymal tissue (such as embryonic urogenital mesenchyme) and into a vascular niche (typically into the sub-renal capsule space) [43,44]. PDXs solve one of the limitations of conventional 2D culture as the prostate cells grow in 3D within the graft. Though PDXs are considered to be the gold standard for many cancers, their use in prostate cancer is more trying as there are low engraftment rates (15-20%) and is expensive [45]. Variability in the engraftment rate of PDXs is further compounded by location of grafting. Sub-renal, subcutaneous and orthotopic grafts have different growth yields but are also associated with increasing skill requirements and technical proficiency [46]. The process is also slow, as once the PDXs are implanted, there is a significant wait for tumour growth (median of 22 months [45]). Furthermore, growth of PDXs is determined by the type of sample engrafted, with metastatic prostate cancer cells more likely to result in successful engraftment than low-risk, non-metastasising cells [47]. In some cases, there can be a lack of serial transplantability of PDXs with engraftments remaining as first-generation [48]. Those that possess the ability of serial transplantation allow researchers to undertake modelling of disease (e.g., LTL331 tumour tissue line [49]). The problem of stochastic mutation accumulation with time, as described above, can lead to significant genotypic shifts away from the patient genome of which the model is designed to represent. Nevertheless, PDXs remain a major tool in the prostate cancer researcher’s armoury. Aside from PDXs, there are other additional complementary approaches to modelling 3D tissue architecture and the main advances have been around in vitro cultures as organoids.

### 2.4. Patient-Derived Organoids (PDOs)

Organoids are in vitro 3D structures which authentically recapitulate the in vivo architecture, molecular make-up and function of the tissue of origin [50]. Organoids differ from spheroids as there is at least pseudo-stratification of cell types within an organoid whereas spheroids are a mass of cells grown as a dense sphere that lack the organisation seen in vivo. Since the 2010s, organoids have emerged as a leading method for in vitro 3D modelling of various organs. Many cell types can be grown as organoids including intestine, stomach, lung and mammary gland [51,52,53]. The development of organoids from human prostate cells has similarly been shown [54,55,56]. This opened the door for a patient focussed, precision medicine approach to in vitro prostate cancer modelling. However, use of patient-derived organoids (PDOs) is limited by similar drawbacks described for primary culture. Whilst there is potential to create numerous prostate cancer lines at various stages of disease progression, organoid generation has low efficiencies and in cases where access to primary prostate cancer tissue/samples is limited this method may not be widely applicable. In established lines, intra-tumour diversification of genetic, epigenetic and transcriptome states evolve in a cell-autonomous fashion [57]. These differences can lead to markedly altered sensitivities to anticancer drugs between even closely related cells derived from the same tumour [57]. 

Failure to translate findings from preclinical models into the patient setting has been one of the factors contributing to the low success rate of anti-cancer drugs making it from the bench to the clinic, and highlights the need for more accurate experimental models [58]. The use of organoid technology to model cancer for drug discovery, drug testing and precision medicine has become of great interest to the cancer field [59]. The fundamental idea behind precision medicine is to tailor medical treatment to the genetic composition of each patient and cancer is a major focus of this initiative [60]. PDOs may be used to more precisely select patients for targeted therapy. 

PDOs have served as a platform for cancer drug screening with studies demonstrating correlation between in vitro drug sensitivities and patient tumour molecular profiles. Gao et al. studied drug response in seven new human prostate cancer organoid lines derived from metastatic and circulating tumour cells expressing disease-specific mutations such as TMPRSS2-ERG fusion, PTEN loss, TP53 loss, SPOP mutations, FOXA1 mutations and CHD1 loss [55]. Correlation of response with the mutational landscape of the tumour was observed following treatment of the tumour organoids with antiandrogen enzalutamide and PI3K inhibitors currently in clinical trials for CRPC. A broad spectrum of AR levels was also observed recapitulating AR-dependent and AR-negative/neuroendocrine phenotypes. However, it was noted that the efficiency of generating organoids from metastatic samples was <20% and reproducibly maintained for only 1–2 months with many cultures overtaken by normal epithelial cells present in the biopsy samples. Van de Wetering et al. established a ‘living’ biobank of colorectal cancer organoids representative of the major molecular subtypes seen in colorectal cancer and performance of a drug screen of 83 compounds including drugs in clinical use detected gene-drug associations that could potentially facilitate personalised therapy [61]. For example, they confirmed resistance to anti-EGFR inhibitors in the setting of KRAS mutant organoids whilst loss-of function mutations of the tumour suppressor TP53 were associated with resistance to MDM2 inhibition. Interestingly, they found RNF43 mutant organoids to be exquisitely sensitive to Wnt secretion inhibitors, potentially identifying a treatment option for patients carrying this mutation. Another drug screen study performed on PDOs discovered novel therapeutic options for endometrial, uterine and colorectal cancer patients [62]. Such studies have paved the way for the development of precision medicine, but the limitations associated with access to primary cultures further highlight the need for additional approaches to improve the generation of PDOs.

Organoid cultures can be expanded long term relatively fast and cryopreserved enabling generation of organoid biobanks, such as the Hubrecht Organoid Technology (HUB) ‘‘living’’ biobank which generates organoids from a vast number of tumour specimens including prostate cancer aiding access to the scientific community [63]. This is a well-characterised library with genome sequencing, expression profiling and drug sensitivity screening data available. Recent studies of living biobanks of patient-derived tumour organoids have reported they retain the genetic landscape of the original tumour and drug responses correspond to patient outcomes. Generation of a “living biobank” of >100 primary and metastatic breast cancer organoid lines captured disease heterogeneity and aided assessment of drug response in a personalised fashion. These efforts highlight the potential of tumour organoid biobanks for high throughput drug screening and precision medicine approaches [61,64,65,66].

However, as advances in models for prostate biology have been hindered by the many challenges associated with primary prostate culture, use of iPSCs presents a promising complimentary approach. Yet, generation of prostate iPSCs has lagged behind that reported from other tissues. Therefore, we describe the significant advances made from using iPSCs to generate models in other systems to demonstrate how the prostate field could take lead from them.

## 3. Human iPSCs for Disease Modelling

In 2006, Takahashi and Yamanaka demonstrated that stem cells with the same characteristics as embryonic stem cells (ESCs) could be generated from adult somatic cells by the simultaneous introduction of just four transcription factors (Oct3/4, Sox2, Klf4, c-Myc), known as Yamanaka factors [67]. iPSCs have the ability to differentiate into almost every cell type of the body, making them powerful tools for disease modelling, regenerative medicine and drug screening [68]. Unlike primary cultures, iPSCs are capable of sustained self-renewal, providing unlimited cell source to investigate diseases, at molecular, cellular and functional levels. Successful reprogramming of prostate tissue and prostate-directed differentiation of iPSCs has been demonstrated, providing scope for such studies in the prostate field [69,70]. Many differentiation protocols towards 2D disease-relevant cell types have been described [71,72,73,74,75,76]. Furthermore, iPSCs are patient-specific and can mimic patients’ phenotypes. In addition to the possibility of genome editing, via knock-in or correction of disease-specific mutations, or knock-out of target genes, iPSCs are becoming one of the preferred choices for disease modelling and personalised medicine approaches.

Despite the huge advances in identifying cell-level phenotypes across a range of diseases, 2D models are restricted to cell culture-based systems and are mostly relevant to the cellular level, failing to represent complex diseases and tumour biology. Due to the significant interest in generating iPSCs from cancer cells to help elucidate the molecular mechanisms of cancer development, various cancer cell lines have been reprogrammed, including prostate cancer [77], melanoma [77,78], breast cancer [79], gastrointestinal cancer [80], chronic myeloid leukaemia [81], glioblastoma [82] and lung cancer [83]. Although reprogramming of cells with cancer genomes is not impossible, it is inefficient and it requires non-integrating methods to avoid increased tumourigenesis and a different combination of transcription factors, alternatively or in addition to the Yamanaka cocktail. Further barriers to generate iPSCs from cancer cell lines and in turn differentiate these cells into the cell-types of interest in 2D or 3D models include cancer-associated mutations, epigenetic modification and high levels of DNA damage [84]. 

### 3.1. Human iPSC-Derived Tissue and Organ Models 

iPSCs have the potential to differentiate into multiple cell lineages and provide a promising source of specialised cells. The differentiation process to the target cell type is a pivotal step in the development of a model. Having gained insights from normal development, many strategies have been explored to guide the direct differentiation of iPSCs mainly by introducing cocktails of growth factors and/or small molecules at defined times and concentrations. In turn, iPSCs have provided models recapitulating single-cell and more complex multi-cell type tissues. For example, generation of human adipocytes (white, brown and beige) from iPSCs, exhibiting mature morphological and functional properties characteristic of in vivo fat tissue, has shed light on adipogenesis and provided a platform for the development of obesity- and diabetes- related therapies [85,86]. TGFβ-driven differentiation of human iPSCs into chondrocytes capable of forming cartilage has opened up new strategies for cartilage tissue engineering, disease modelling and pharmacological drug studies for osteoarthritis [87]. In contrast, the adult human heart harbours several different cell types. Functional cardiomyocytes generated from iPSCs hold significant promise as an autologous cell source for cardiac repair [88]. However, cardiomyocytes comprise only 25% of all cardiac cells even though they occupy approximately 75% of normal myocardial tissue volume, whilst atrial and ventricular cardiomyocytes have distinct action potentials [89]. As such, presently, generation of a fully beating heart is a long distance away. Although such studies have been informative, they also have limitations mainly that differentiation strategies are based on 2D cultures. 

More recently, iPSC-derived cancer organoids have been described. Using standard iPSC differentiation techniques coupled with recent advances in bioengineering, xenotransplantation and genome editing, iPSCs present new opportunities for the study of human cancer [90]. These methods increase the generation of cells to undertake high throughput assessment that have otherwise been previously challenging, such as wide scale in vitro organoid drug testing and organ-on-chip assays. In this context, 3D iPSC-derived organoids (iDOs) have now been generated for multiple organs due to their resemblance to stratified cell organisation and organ structure, which better represents human physiology and development (Table 2). iDOs provide new tools for the study of human development, disease and drug testing from which the prostate field can learn from and are successfully generated in nearly every differentiation run.

#### 3.1.1. Cerebral iDOs

The groundbreaking work described by Yoshiki Sasai and colleagues, in a seminal paper [103], demonstrated how serum-free suspension culture of embryoid bodies, and the temporal addition of small molecules to generate forebrain neural precursors, led to an in vitro model of brain-like organoids [91]. These 3D models, called cerebral organoids, contained regions that resembled various compartments of brain regions, such as cortical-like regions, similar to that of the human embryo cortex. The addition of temporal inductive signals to these 3D models in subsequent studies, was shown to drive dorsal and ventral fore-brain differentiation [104], containing a variety of cell types present in the human cerebral cortex [105]. Ventricular-like zones of cerebral organoids containing neural stem cells expressed markers of deep- and superficial-layer neurons and outer radial glial cells, only present in humans [106]. This has brought about a huge interest in organoid technology as a means to investigate human-specific conditions and the development of the human brain [92]. 

#### 3.1.2. Retinal iDOs

Further work from Yoshiki Sasai described the self-organisation of pluripotent stem cells to form optic cup structures, via temporal application of small molecules [93], displaying features of multi-layered retinal architecture that resembled foetal human neural retinas. Importantly, some iPSC-derived retinal organoids have been shown to be light responsive, with immature signals simulating those observed in the neonatal retina [107]. Retinal organoids have been used to interrogate and treat a variety of eye-degenerating conditions as well as cancer [94,95,108].

#### 3.1.3. Intestine iDOs

Intestine organoids can also be derived from iPSCs in a 3D culture system by the use of extracellular support matrix and culture medium supplemented with pro-intestine growth factors [96]. These organoids contained defined 3D structures reflecting the villus and crypts of the small intestine and were capable of self-renewal and self-organisation for prolonged cultures [109,110]. iPSC-derived colonic organoids have been used for the modelling of colorectal cancer [111].

#### 3.1.4. Liver iDOs

iPSC-derived liver organoids have been generated by co-culture of iPSCs, mesenchymal and endothelial cells. Liver buds were formed by self-organisation and cell-to-cell contact combined with paracrine signalling, resulting in induction of hepatic genes and expression of bile salt export pumps [97]. More recently, generation of liver organoids on a 3D perfusable chip has been reported [98]. These organoids had higher cell viability and expressed endodermal and mature hepatic genes. Transplantation of liver buds in mouse models of liver failure partially rescued hepatic function [112]. 

#### 3.1.5. Kidney iDOs

iPSCs can also be differentiated into kidney organoids using a mesoderm induction step followed by 3D culture to promote self-organisation leading to organoid formation. These kidney organoids contained segmented nephrons connected to collecting ducts, surrounded by renal interstitial cells. Proximal tubules within the organoids were able to carry out endocytosis, as evidence of functional maturity [99]. Kidney organoids can be used as a platform to develop new drugs to treat chronic kidney disease [113].

#### 3.1.6. Lung iDOs

Lung organoids have also been successfully generated and used to study lung development and disease [114,115]. Lung organoids can be derived from iPSCs using an endoderm induction step followed by the addition of key defined growth factors, and further passage into a 3Ds system using extracellular support matrix, in order to promote branching morphogenesis, growth, and alveolar cell formation [96,100,101,102]. Those airway progenitors contain cell types and structures similar to those of bronchi/bronchioles of early lung development and express alveolar-cell markers. Importantly, the iPSC-derived models have been benchmarked with human foetal tissue and the transcriptomics of organoids have been shown to be similar to that of foetal lung [102].

## 4. Emerging Approaches in Preclinical Prostate Cancer Research

### 4.1. Transformation of Primary Prostate Cells

In a seminal paper, it was shown that primary benign human basal prostate epithelium can initiate prostate cancer in immune-deficient mice and that the derived tumours realistically recreate histology of in situ human prostate cancer [116]. This has been the basis for an interesting strategy to overcome some of the problems of primary prostate cancer culture, where researchers can now transform easier to grow benign prostate epithelium to generate prostate cancer organoids [117]. These studies lay the platform for a new paradigm, where benign cells are converted into “designer” cancers harbouring specific mutations of interest. These can be repeatedly generated whilst faithfully maintaining the genotypes of interest avoiding the ever evolving subclonal progressions affecting long term culture of primary derived cancers [57]. Despite the promise of this “tumour engineering” approach there are limitations to maintaining even benign prostate epithelial cultures and a more ready supply of cells to manipulate are ideally required. In this respect, the emergence of easy to expand and immortalised iPSCs and the ability to differentiate these in the tissue type of interest offers a new way forward. 

### 4.2. Prostate iDOs

Recently, a high throughput model of generating human prostate organoids from iPSCs has also been described, involving co-culturing iPSCs with rodent urogenital sinus mesenchyme (UGM). This simple differentiation protocol results in glandular structures in vitro that faithfully mimic prostate tissue histology and express key prostate markers such as AR, prostate specific homeobox protein NKX3.1 and secretory prostate specific antigen (PSA) [69]. This approach built on previous data showing the generation of prostate tissue in xenograft studies from ESCs [118]. Differentiation from iPSCs avoids many ethical and regulatory restrictions relating to ESCs and enable greater access to organoid generation to groups worldwide culture [119,120]. Previous in vitro human prostate organoid approaches, from either tissue-derived cells or ESCs, do not recreate the full breadth of in situ prostate differentiation as they do not contain neuroendocrine cells particularly relevant in light of emerging data showing that neuroendocrine differentiation drives treatment-resistant prostate cancer [56,121,122]. Additionally, it would be of interest to determine whether following maturation of prostate iDOs there is a switch to a somatic stem cell mode of homeostasis, identified by the presence of DLK1-enriched basal stem cells, to sustain long-term culture [123]. High-throughput iPSC-derived human prostate tissue generation provides unparalleled scope for in vitro disease modelling and drug discovery without the constraints of tissue accessibility and long-standing difficulties associated with primary culture. 

### 4.3. Genome Editing Technology and Precision Medicine 

Genome editing technology has emerged as an extremely powerful tool that can greatly advance organoid-based research for the development of better targeted therapies [124]. CRISPR-Cas9 genome editing induces double-stranded DNA breaks at specific loci adjacent to a protospacer-adjacent motif (PAM) using a complementary single-guide RNA sequence (sgRNA) and Cas9 endonuclease [125]. DNA repair then takes place by either non-homologous end joining, resulting in insertions/deletions (INDELs) or homology-directed repair with a donor template. In 2013, Schwank et al. reported the first successful therapeutic CRISPR-Cas9 gene editing in human tissue, by correcting the CFTR locus in intestinal organoids from patients with cystic fibrosis (CF), making CF treatment a possible reality [126]. Since then, CRISPR-Cas9 has further been used to reproduce genetic mutations that occur in cancers including prostate cancer. In 2017, for the first time, the use of CRISPR/Cas9 to create endogenous gene fusions in organoids was reported [127]. Mouse prostate organoids were modified to carry the TMPRSS2-ERG fusion, a genetic alteration present in more than 50% of prostate cancers that leads to high ERG expression driven from the androgen-responsive promoter of the TMPRSS2 gene [127,128,129]. Previously this fusion had been modelled by artificial ERG overexpression and studied in human prostate cancer cell lines and mouse models, but this approach for the first time allows investigation of its effect in a wildtype background [127,130].

## 5. Final Remarks

Effective treatment development for prostate cancer is hampered by the lack of patient-specific in vitro models that accurately recapitulate this disease. The prostate-derived iPSC generation of human prostate tissue both in vivo and in vitro is a new complimentary approach to established primary culture and PDX models [69]. Together with genome editing technologies such as CRISPR/Cas9, this model opens up new avenues to recreate the genetic make-up of individual patients and correlate drug sensitivity in vitro in a personalised fashion. Introduction of patient-specific mutations into iPSCs to generate “designer” cancer organoids could lead to the creation of organoid biobanks covering the spectrum of prostate cancer mutations and facilitate the design of powerful screening platforms. Proof of concept is already established showing that benign prostate cells can be transformed into prostate cancers [116]. This approach would overcome a major problem with the low efficiency of prostate cancer organoid culture, issues with significant genetic drift associated with long-term primary culture and has the ability to reproduce, with high fidelity, isogenic cultures time after time. In the future, routine genomic testing would define patient-specific profiles and the biobank would provide that reference genotype for new drug testing or known sensitivity to pre-tested standards of care to allow clinicians to tailor treatments options to improve outcomes in cancer patients.

## Figures and Tables

**Table 1:** Advantages and disadvantages of current models of prostate cancer.

Model	Advantages	Disadvantages
Cancer cell lines	Easy and cheap to grow; Useful for basic science; High throughput drug screening	Limited to 2D; Mutation accumulation over time; Limited number available
Primary cells	Derived from patients; Initial drug studies; Use for PDXs, PDOs and iPSCs	Difficult to grow; Tissue accessibility; Limited to 2D; Mutation accumulation over time
Patient-derived xenografts (PDXs)	Retain 3D tissue architecture; Intact endocrine system; Disease stage-specific models available	Time consuming and expensive; Low engraftment efficiencies; Mouse has deficient immunity and different microenvironment
Patient-derived organoids (PDOs)	Retain 3D tissue architecture; Histological and molecular resemblance to tissue of origin; Drug testing responses more accurate	At present only established from aggressive prostate cancer specimens; Low establishment rate; Lack microenvironment and immune influence
iPSC-derived organoids (iDOs)	Retain 3D tissue architecture; Unlimited source of iPSCs; Isogenic lines; Gene editing to introduce patient-specific mutations; High throughput drug screening; ‘avatar’ for precision medicine	Lack microenvironment and immune influence

**Table 2:** Generation of iPSC-derived organoids.

Tissue/Organ	Method	Key Small Molecules	References
Brain	Self-organisation by embryoid bodies formation, and the addition of temporal small molecules	IWR1 and SB431542	[91,92]
Eye	Self-organisation by embryoid bodies formation, and the addition of temporal small molecules	BMP4 and IGF1	[93,94,95]
Intestine	Extracellular support matrix and culture medium supplemented with pro-intestine growth factors	Activin A, WNT3A and FGF4	[96]
Liver	Co-culture of iPSCs with mesenchymal and endothelial cells followed by self-organisation by cell-to-cell contact or self-organisation by embryoid bodies formation on 3D perfusable chip	Activin-A, bFGF and HGF	[97,98]
Kidney	Mesoderm induction step followed by self-organisation in 3D culture	CHIR99021 and FGF9	[99]
Lung	Endoderm induction, addition of temporal small molecules and culture in extracellular support matrix or transwell inserts	Activin A, Noggin, SB431542, SAG, FGF4, CHIR99021 and FGF10	[96,100,101,102]
Prostate	Endoderm induction step and co-culture of iPSCs with rodent urogenital sinus mesenchyme (UGM), followed by self-organisation by cell-to-cell contact in extracellular support matrix	Activin A, EGF, R-spondin1, Noggin, and A83-01	[69]

## Abbreviations

AR	Androgen receptor
CRPC	Castration-resistant prostate cancer
CRPC-NE	Castration-resistant prostate cancer neuroendocrine
CF	Cystic fibrosis
ESC	Embryonic stem cells
iPSCs	Induced-pluripotent stem cells
iDOs	iPSC-derived organoids
PAM	Protospacer-adjacent motif
PDOs	Patient-derived organoids
PDXs	Patient-derived xenografts
PSA	Prostate-specific antigen
sgRNA	Single-guide RNA sequence
UGM	Urogenital mesenchyme
